# Deep neck abscess as the predominant initial presentation of carcinoma of unknown primary: A case report

**DOI:** 10.3892/ol.2014.1835

**Published:** 2014-01-28

**Authors:** WEI-TING CHEN, JUI-WEN LEE, KUN-WEI HSIEH, RONG-FENG CHEN

**Affiliations:** Department of Otolaryngology, Kaohsiung Armed Forces General Hospital, Kaohsiung 802, Taiwan, R.O.C.

**Keywords:** carcinoma unknown primary, deep neck infection

## Abstract

Malignancies, which present as deep neck abscesses are uncommon and may result in a delayed diagnosis or potentially a misdiagnosis. The present study describes a patient who exhibited a deep neck abscess as the initial manifestation of carcinoma of unknown primary (CUP). The aim of the present study was to raise awareness of this unusual presentation of CUP and emphasize the importance of repeating targeted fine-needle aspiration cytology or biopsies in patients presenting with a deep neck abscess suspicious for malignancy.

## Introduction

Deep neck space abscesses are common and the majority of cases originate from infections or trauma to the head and neck structures (1–5). A neck abscess or cervical cellulitis as the initial presentation of carcinoma of unknown primary (CUP) is rare. CUP is an uncommon malignancy and is defined as the presence of metastatic cancer without an identifiable primary origin (6–8). To the best of our knowledge, the presentation of CUP as a deep neck abscess has not been previously reported in the English literature. The aim of the present case was to avoid potential pitfalls in the management of deep neck abscesses. Furthermore, it was proposed that for the purpose of accurate diagnosis, fine-needle aspiration of neck masses should be conducted promptly according to the age of the patients and the risk factors that are typically observed in malignancies.

## Case report

### Case presentation

A 71-year-old female, with a betel nut-chewing habit and a history of hypertension, presented with a fever and a painful swelling on the left side of the neck which had both lasted for three days. The neck mass, which was diagnosed as a cervical lymphadenitis at a local clinic, had been present for one week, had progressively increased in size and had become red. The physical examination showed that the patient had a temperature of 38.3°C and presented with a tender mass (size, 3 cm) and skin erythema on the left side of the neck at level II-III. The total white cell count was 18.7×10^9^/l and the C-reactive protein value was 12.3 mg/dl (normal value, <0.5 mg/dl). No additional clinical abnormalities, such as transnasal fiberoptic laryngoscopy, were identified as a result of the head and neck examinations. The patient provided written informed consent.

### Imaging and diagnosis

**Figure 1:**
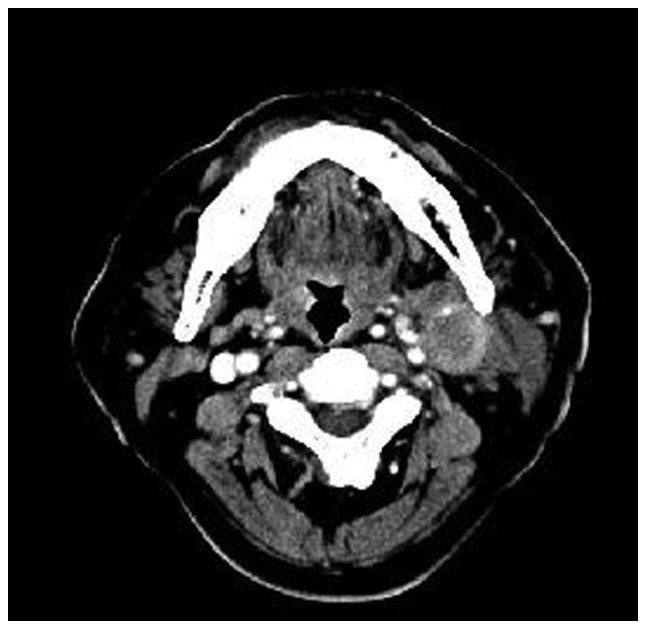
Axial, contrast-enhanced computed tomography shows a hypodense and cystic lesion with ring enhancement, located in the deep neck-space on the left side of the neck.

**Figure 2:**
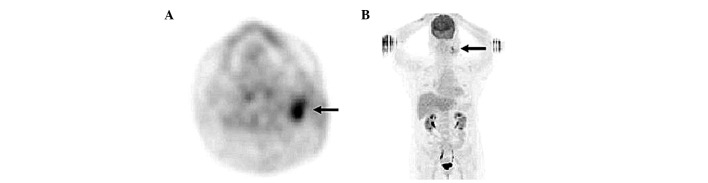
F-18-fluorodeoxyglucose (FDG) positron emission tomography (PET) images prior to the modified radical neck dissection and radiotherapy treatment revealed no definite FDG-avid lesion, with the exception of the left level II area of the neck. (A) Axial FDG-PET. (B) Maximum intensity projection FDG-PET. The arrows identify the cervical lymph node metastasis and the standardized uptake value was 5.7.

An ultrasonographic scan showed a hypoechoic and heterogeneous deep abscess between the left sternocleidomastoid muscle and the common carotid artery. A contrast-enhanced computed tomography scan of the neck was performed and a hypodense predominantly cystic lesion, exhibiting ring enhancement over the left carotid space, was observed (Fig. 1). The pus culture developed *Staphylococcus aureus* and the cytological examination via fine-needle aspiration revealed the presence of non-malignant inflammatory cells alone. Following adequate control of the infection, the patient became afebrile and the skin erythema ameliorated. However, the neck mass remained present and the possibility of a malignancy could not be excluded due to the patient regularly chewing betel nut and a concern regarding the lateral cervical cystic lesion that the patient exhibited. Therefore, an additional fine-needle aspiration of the lesion was conducted and the cytology revealed malignant epithelial cells, which were consistent with squamous cell carcinoma. The diagnostic procedures were conducted to characterize the occult carcinoma, although the results of the abdominal ultrasonographic scan, chest radiography and the bone scintigraphy were all observed to be normal. The primary fluorodeoxyglucose (FDG) uptake site was detected by F-18-FDG positron emission tomography in the lymph nodes of the left-sided level II area of the neck, with a maximum standardized uptake value of 5.7 (Fig. 2). Therefore, the patient was diagnosed as exhibiting a CUP with lymph node metastasis in the neck, in addition to a deep neck abscess.

### Patient outcome

The patient subsequently underwent a left side modified radical neck dissection, which was followed by radiotherapy treatment. The patient survived and showed no indication of recurrence within the five-year follow-up.

## Discussion

Neck abscesses and deep neck infections are common diseases, which may arise from various head and neck regions, including the teeth, adenotonsillar tissues, the nasal cavity, pharynx, paranasal sinuses and the salivary glands. Odontogenic and tonsillar infections are the predominant causes; however, malignancies may occasionally present with one of the aforementioned deep neck infections. In previous studies, sporadic cases of metastatic carcinoma presenting as deep neck abscesses from certain head and neck regions were reported, such as the paranasal sinus, nasopharynx, tonsil, tongue base, pharynx, larynx, thyroid and parotid glands (1–5). However, these were not related to CUP.

CUP was identified to account for ~3–5% of newly diagnosed cancer patients in the Swiss population (6), representing a heterogeneous group. It is defined as the presence of metastatic cancer without an identifiable primary origin, based on obtaining a detailed medical history and conducting precise clinical examinations, imaging of anatomic sites and diagnostic investigations (7). Although patients who exhibit cervical lymph node metastasis often present with a primary cancer site in the head and neck region, no primary tumor was identified in a previous study, despite extensive diagnostic workups in ~2% of the patient group that was observed (8). In the present case, there was no indication of the primary tumor. Thus, it was hypothesized that the tumor had receded as a result of spontaneous regression by apoptosis or immune-modulated destruction, following metastasis to the local lymph nodes (9,10). Alternatively, the tumor may have been too small for accurate sampling (11). However, the possibility that the tumor cells may have originated from benign epithelial inclusions in the lymph node of the neck and were destroyed by the growth and spread of the tumor (12) could not be excluded. Moreover, the occurrence of ectopic epithelium in the lymph nodes is uncommon and embryonic admixing is the most likely explanation (12).

Malignant lymph node metastasis, which presents as a deep neck abscess or cervical cellulitis, is rare and may be due to the relatively effective vascular supply to the head and neck region (1). The causes and predisposing factors for this type of deep neck infection remain unknown; however, it has been hypothesized that the invariably infected ulcer of the primary tumor is a potential source of the abscess-forming bacteria, which drain into the lymph nodes (2). Conversely, the center of a large malignant lesion may be susceptible to infection due to tumor necrosis, which results from an insufficient vascular supply (3). The organism most commonly observed in a tumor abscess is *Staphylococcus aureus* (2).

The infections that coexist with the malignancy complicate the clinical observations and may lead to a delayed diagnosis. This is due to the infection superimposing on the malignant process, which reduces the likelihood of obtaining a biopsy that exhibits a good representation (4). In patients with a high risk of head and neck squamous cell carcinomas, such as smokers, alcohol drinkers, those that chew betel nut and elderly patients, a high index of suspicion must be maintained even when the initial cytology is benign. Furthermore, age may provide an indication into the differential diagnosis of the patients that exhibit a deep neck infection. Previous studies have identified that the average age of patients (range, 40–74 years; median, 64 years), with deep neck infections and malignancies, was two decades older than that of the patients exhibiting simple pyogenic deep neck infections. These commonly occur in a younger population, aged between 20 and 40 years (5), and the clinical symptoms of the patients that were observed were similar to those of the patients exhibiting a simple pyogenic deep neck infection. Therefore, establishing a careful follow-up procedure after initial treatment is recommended. This may enable the detection of possible occult carcinomas in patients who exhibit an obscure etiology of infection or those who present typical risk factors for squamous cell carcinoma. Moreover, the current case demonstrated a requirement for the careful review of cytological specimens, as in the present study, the precise diagnosis was determined following the repetition of the cytologic evaluation. It is likely that the result of the initial pathological examinations of the abscess aspirates or the abscess wall may be negative for malignancy when there are abundant inflammatory cells; thus, the presence of a marginal number of atypical clusters of cells may be ignored. Furthermore, the clinical presentation may influence the diagnosis. Although a benign pathology may be observed where the patient exhibits a resolved neck abscess, these cases should be carefully followed up as a malignancy may manifest during the convalescence of the abscess.

In the management of these cases of deep neck abscesses associated with malignancy, a definitive treatment for the tumor and an appropriate treatment of the abscess are required. In addition, detection of the primary site is critical as this may result in patients receiving site-specific treatment with a favorable prognosis. The majority of primary sites are able to be confirmed pathologically, shortly following the diagnosis of a cervical metastasis. Therefore, detailed medical history-tracing, careful investigation and pathological examination of tissues obtained from fine-needle aspiration or surgical biopsy in the suspected malignancy area, are important to reduce the time it takes to identify the primary malignant origin. Although the primary origin of the malignancy in the present study was not identified, a good recovery was observed following the modified radical neck dissection and the radiotherapy treatment.

In conclusion, malignancies may present as deep neck abscesses and patients may, therefore, require a careful examination, particularly those who exhibit a high risk of head and neck carcinoma.
